# Effect of Contacting Surface on the Performance of Thin-Film Force and Pressure Sensors

**DOI:** 10.3390/s20236863

**Published:** 2020-11-30

**Authors:** Ka Po Maggie Tang, Kit Lun Yick, Pui Ling Li, Joanne Yip, King Hei Or, Kam Hong Chau

**Affiliations:** 1 Institute of Textiles and Clothing, The Hong Kong Polytechnic University, Hung Hom, Hong Kong; tc.maggie@connect.polyu.hk (K.P.M.T.); puiling-sabrina.li@polyu.edu.hk (P.L.L.); joanne.yip@polyu.edu.hk (J.Y.); 20032498R@connect.polyu.hk (K.H.O.); edward.chau@connect.polyu.hk (K.H.C.); 2 Laboratory for Artificial Intelligence in Design, Hong Kong Science Park, Taipo, Hong Kong

**Keywords:** pressure comfort, force sensor, pressure sensor, sensor evaluation, hardness, temperature

## Abstract

Flexible force and pressure sensors are important for assessing the wear comfort of tightly fitting apparel. Their accuracy and repeatability depend on the sensor itself and the contacting surface. Measurements of the contact pressure on soft surfaces like human skin tend to be erroneous, which could be due to incorrect sensor calibrations. This study aims to examine the effects of human body parameters such as the hardness and temperature of the contacting surface by using a custom-made calibration setup and investigating the incorporation of rigid discs on the sensor surface. Two commercial force sensors, FlexiForce and SingleTact, and one pressure sensor, Pliance X, are used in the investigation. The findings reveal that adding rigid discs on both sides of the force sensors improves their sensitivity. Systematic calibration has been performed on the surfaces with different temperatures and hardness. The results show that FlexiForce and Pliance X tend to be affected by the changes in surface temperature and surface hardness. Prolonged testing time shows that the time dependence of SingleTact and Pliance X sensor is lower, which suggests that they are more suitable for lengthier evaluations in which interface pressure is exerted on the human body. In brief, sensor attachment and proper calibration should be thoroughly considered before using sensors for applications on soft surfaces, like the human body.

## 1. Introduction

Pressure garments or elastic bandages are used in pressure therapy, for instance, to treat hypertrophic scars following a burn injury. They help to control the formation of excess wound collagen and suppress the growth of hypertrophic scars [1,2,3]. The pressure applied from such garments or bandages onto a hypertrophic scar normally ranges from 10–25 mmHg (i.e., 1.33–3.33 kPa) [4,5]. Pressure garments have also been used to treat chronic venous disorders and venous leg ulcers [6,7]. The pressure applied for these conditions is however much higher but still does not exceed 70 mmHg (i.e., 9.33 kPa) [8]. Controlling the applied pressure is important to treat various conditions and provide a good wear condition, and the appropriate amount of induced pressure can contribute to improving the product design. Insufficient pressure means that the treatment is less effective whilst excessive pressure can lead to tissue damage, or might even require amputation in severe cases [9]. Nowadays, thin-film force and pressure sensors have been broadly utilized to measure the interfacial pressure of intimate apparel [10,11,12,13], compression stockings [8,9,14], sports compression garments [15,16], a posture correction girdle [17], and pressure garments for burn treatment [1,3,18], as well as the plantar foot pressure in gait analyses [19,20]. The thin-film force and pressure sensors help to control the exact pressure applied and optimize the fit of the end-products.

Pliance X sensors (Novel Electronics, Germany), which are capacitive sensors, have been widely used in low interfacial pressure applications [1,2,21]. These sensors are sensitive, but very costly. Alternatively, force sensors like FlexiForce^®^ (Tekscan, Boston, MA, USA) and SingleTact sensors have been developed to measure the interfacial pressure when force is applied to a known surface area [22]. These force sensors are cost-effective and designed for low force measurements.

Many researchers have investigated the performance of various force sensors for the aforementioned applications [7,8,23,24]; however, no study has provided a comprehensive evaluation with the same testing conditions. In addition, the sensors are mostly calibrated on a flat rigid surface by applying different known weights [6,15,23]. This may be acceptable for applications such as shoe insoles where the contacting object is relatively flat. However, for applications where the sensor is placed onto the human body which is compliant and has contours, the pressure might not be evenly distributed across the sensor surface which would result in erroneous measurements. In view of this, some researchers have tried to simulate different end-use conditions and measured surfaces with different geometries [9,14]. Surfaces with different curvatures have been one of their main focuses. For instance, Ferguson-Pell et al. [22] used the FlexiForce^®^ sensor to measure pressure on rigid cylinders with different radii and found that changes in the curvature affect both the offset value and sensitivity of the sensor. This agrees with the findings in Komi et al. [25], and Buis and Convery [26] where changes in the surface curvature lead to an increase in measurement error. Surface curvature also leads to the problem of sensor bending. To reduce the likelihood of sensor bending when a sensor is placed onto a curved surface and avoid saturation of the sensor from punctual applied loads, researchers have attempted to remedy this problem by fixing attachments onto the sensor. For instance, Jensen et al. [27], Hall et al. [28], and Flórez and Velásquez [29] fixed a dome onto a sensor to increase its rigidity and thereby eliminate bending when the sensor comes into contact with a curved surface. Likitlersuang et al. [30] evaluated the possibility of adding a thin rigid disc (plastic disc with a thickness of 1.02 mm) on top of the sensor or underneath the sensor, and confirmed that adding a thin rigid disc between the sensor and human body and calibrating done on a compliant surface help to reduce measurement errors. All of these studies suggest that the sensor surface should be properly selected for compliant surface measurements and prove the importance of proper calibration. However, Likitlersuang et al. [30] conducted their tests on the forearm of their subject, which might potentially result in inaccuracies in the sensor readings, and therefore more systematic testing should be carried out.

Apart from the sensor itself, the calibration method of a sensor affects the accuracy of the measurement results. Most of the previous studies apply a series of dead weights to the sensor repeatedly which range from 0 to 55 g [31], 2 to 52 g [16], 370 g to 11.2 kg [25], and 50 g to 1 kg [23]. This process is, however, quite time-consuming and it can be difficult to place the weight in the same position of the sensor [23]. Most importantly, they did not calibrate the sensor or perform testing on soft tissues that are similar to those of the human body, both of which potentially affect the accuracy of the final results. On the other hand, Khodasevych et al. [7] and Likitlersuang et al. [30] did consider the hardness of the contacting object. The former chose to use silicone with a hardness of 10 Shore A as the contacting object whilst the latter used a polyurethane disc with a hardness of 60 Shore A to simulate the soft skin at the body/device interface for pressure evaluation. However, both failed to provide much information on how the hardness is related to human body tissues. The hardness of the human body considerably varies between individuals and different areas of the body; see Appendix A. Therefore, the effect of the hardness of the contacting object on the interface pressure should be systematically studied, thus providing a reliable tool for measurements of low interface pressure between the human body and different surface hardness.

Normally, calibration is performed at room temperature. However, the effect of different temperatures on the sensing performance of sensors has not been systematically evaluated. To date, no study has provided a comprehensive analysis of the effect of temperature on sensor performance. When a sensor is placed onto human skin, the temperature of the contacting surface is around 33 °C. The local skin temperature can vary with activity intensity and climate. The temperature of human skin ranges from 32.6 °C at an environmental temperature of 25 °C but increases to 35.5 °C at an environmental temperature of 37 °C [32]. In this case, the temperature difference between the skin surface and calibrated condition can be higher than 10 °C.

**Figure 1:**
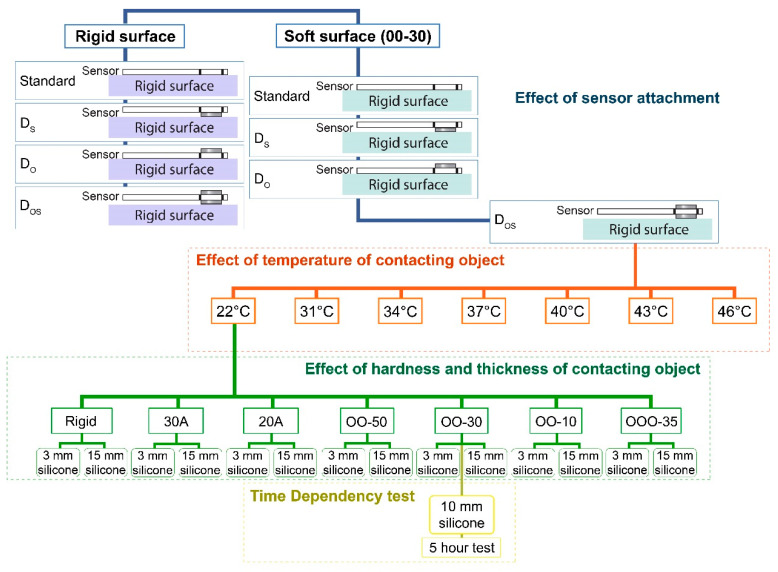
Experimental plan. Notes: D_O_: disc is placed between applied force and sensor; D_S_: disc is placed between sensor and contacting object; D_OS_: one disc is placed between the applied force and sensor and another disc is placed between sensor and contacting object.

Tightly fitting garments, including sportswear and intimate apparel, have direct contact with the skin, so more diligence is needed to measure the pressure comfort due to variations in the body geometry and hardness and surface temperature of the skin [7]. In light of this, the objectives of this study are to (i) develop a simple, repeatable, and representative experimental protocol for dynamic force/pressure sensor calibration; (ii) study the effect of sensor attachment on the sensitivity of the sensor, and linearity of the calibration curve on rigid surface; (iii) investigate the effect of surface temperature of the contacting object on the reading of the sensor; (iv) examine the effect of the surface hardness on the reading of the sensor; (v) quantify the result discrepancies if the sensors are calibrated in standard conditions but used in other conditions (e.g., calibrated on rigid surface at room temperature but used on skin); and (vi) determine the sensor drift against time. Details of Objectives (ii), (iii), (iv), and (vi) are illustrated in Figure 1.

## 2. Materials and Methods

### 2.1. Sensor Specifications

**Figure 2:**
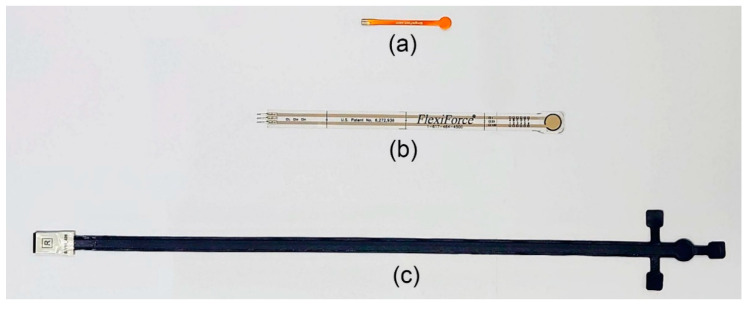
Evaluated force and pressure sensors: (**a**) SingleTact force sensor, (**b**) FlexiForce^®^ sensor and (**c**) Pliance X pressure sensor.

Three types of commercially available sensors, which are suitable for low pressure measurements, were investigated under controlled laboratory conditions and are shown in Figure 2. They include the FlexiForce and SingleTact force sensors and Pliance X pressure sensor. Their specifications are summarized in Table 1.

**Figure 3:**
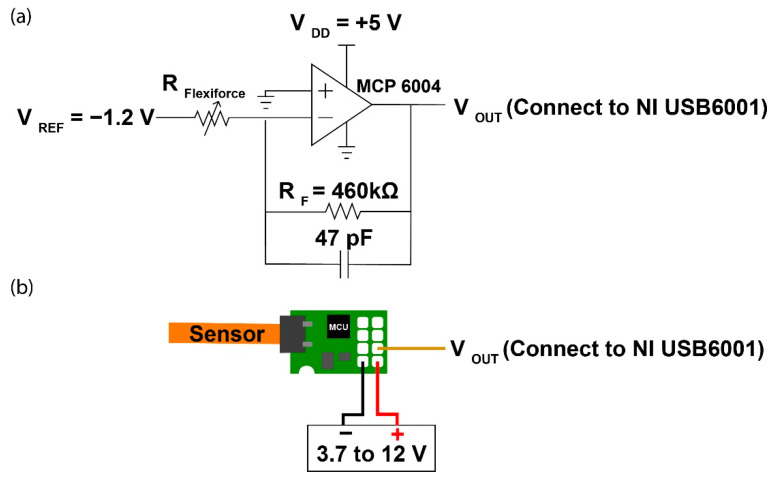
(**a**) Circuit for FlexiForce^®^ sensor, and (**b**) Pinout diagram of SingleTact sensor.

The FlexiForce sensor is made of two layers of polyester film. A layer of conductive material is applied onto each layer of polyester film followed by a layer of pressure sensitive ink. This sensor is a variable resistor and connected to an inverted operational amplifier circuit, as shown in Figure 3a, which converts the measured resistance to a voltage [33]. The electrical resistance is reduced with higher applied pressure due to the displacement of the ink in the sensor during loading. The sensitivity of the sensor can be adjusted by changing the reference resistance (R_F_), in which a lower R_F_ will result in reduced sensitivity of the sensor (i.e., increased force range). With reference to our desired pressure range, an R_F_ of 460 kΩ was selected. The V_OUT_ is connected to USB6001 (accuracy: 6 mV; ADC resolution: 14 bit; user-sampling rate: 20 samples/s) from National Instruments Corp. for data logging.

The SingleTact sensor is a capacitive force sensor which consists of a sensor dielectric that is laminated in two layers of electrodes [34]. The signal is converted by the supplied I2C board which is connected to the USB6001 converter for data logging. The manufacturer-recommended pinout diagram is shown in Figure 3b. The analog output swings from 0.5 V (no force applied) to 1.5 V, but the output in the zero force condition is arbitrary (fluctuating at around 0.5 V). The absolute voltage which corresponds to the applied force can be calculated by subtracting the zero force voltage from the measured voltage as follows:

(1) $${\mathrm{Voltage}}_{\mathrm{force}}={\mathrm{Voltage}}_{\mathrm{measured}}-{\mathrm{Voltage}}_{\mathrm{zero}\;\mathrm{force}\;}$$

The Pliance X sensor is composed of two parallel plates separated by a dielectric medium. The change in capacitance occurs because of the variation in the distance between the two plates. The software provided was used to automatically record the output and used pressure as the unit.

As shown in Table 1, the FlexiForce and SingleTact sensors are particularly thin and deform easily. In order to avoid sensor bending, discs were attached to the sensors and four sensor conditions were tested as follows. Standard corresponds to the only sensor condition where no disc is adhered onto the sensor surface. D_O_ (Disc-outside) corresponds to the condition where a disc is placed between the applied force and the sensor. D_S_ (Disc-skin) corresponds to the condition where the disc is placed between the sensor and the contacting object. D_OS_ (Discs-outside-and-skin) corresponds to the condition where one disc is placed between the applied force and the sensor, and another disc is placed between the sensor and the contacting object. The discs are made of 6061 aluminum with a thickness of 0.5 mm. As for the FlexiForce^®^ sensor, aluminum discs that are 8 mm in diameter (70–85% of the sensing area as suggested by the manufacturer) are placed on top of the sensor whereas the bottom is 13 mm in diameter. The disc at the bottom is larger than the sensing area to ensure that the force applied is concentrated within the sensing area. For the SingleTact sensor, the diameter of the aluminum disc adhered to either side of the sensor is equivalent to the active sensing area (8 mm). Double-sided tape was used to adhere the disc onto the sensor.

### 2.2. Testing Apparatus

**Figure 4:**
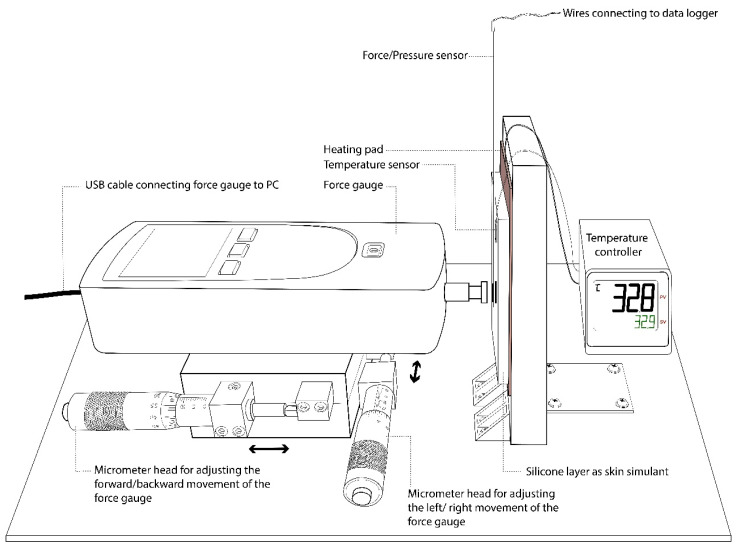
Experimental setup for sensor calibration.

Figure 4 illustrates a custom-made apparatus designed to load the sensor under a simulated skin condition. Unlike testing with dead weight calibration, loading can better approximate dynamic wear conditions; for example, short-term changes in pressure during physical activity or respiration. An acrylic plate with a thickness of 1 inch and resembles a vertical contacting surface, was firmly adhered to the base plate. For the conditions where the contacting surface is soft (like human skin), a layer of silicone (Smooth-On, Inc. (Macungie, PA, USA)) with different levels of hardness was adhered onto the acrylic plate. The hardness of these silicone samples is summarized in Table 2. For the conditions with a heated contacting surface, a heating pad which was connected to a 12 V power supply, was placed underneath the silicone layer and a resistance temperature detector (RTD) sensor (PT100, 2 mm × 10 mm, accuracy: 1/3 DIN) from RS Pro was placed on top of the silicone layer. The heater as well as the RTD sensor were connected to the E5CC digital temperature controller (ORMON, Hoffman Estates, IL, USA) to monitor the temperature changes and turn the heater on or off. As for the force/pressure sensor, it was affixed to the contacting surface by using 3M Micropore tape.

On the other hand, a force gauge (Model no.: Chatillon DFS II, 10 lb. Accuracy: ± 0.1%) (AMETEK, Inc., Bowen, PA, USA) was mounted on a linear translation stage which allows for movement of the XY axis. Two micrometer heads (Accuracy: ± 20 µm) were used to control the left/right and forward/backward movements of the force gauge. After the position of the loading surface was found to be in agreement with that of the sensor, it was ready for the calibration process.

**Figure 5:**
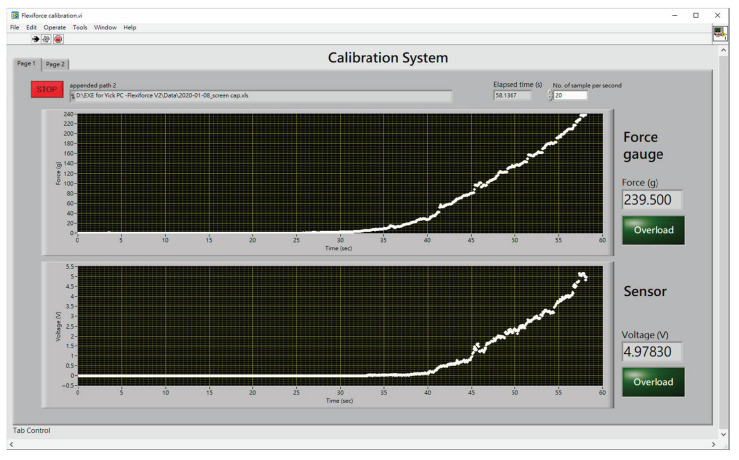
Control panel for sensor calibration.

By slowly moving the force gauge towards the force/pressure sensor at a speed of around 0.4 mm/min, the load applied as well as the force/pressure exerted onto the sensor were recorded. The LabVIEW 2015 program was used to record the data from both the force gauge and sensor. The control panel for the sensor calibration is shown in Figure 5.

### 2.3. Percentage Error

The percentage error of the sensor output was calculated as the difference between the applied (by force gauge) and measured (by sensor) pressures divided by the applied pressure. Equation (2) shows the calculation of the percentage error under a specific pressure level. Equation (3), on the other hand, shows the calculation of the mean percentage error under a specific range of pressures.

(2) $$Percentage\;Error\;=|/P_{ref}\times 100\%$$

(3) $$Mean\;Percentage\;Error=\frac{1}{N}\overset{N}{\underset{n=1}{\sum }}\left[\frac{\left|P_{sens}\left(n\right)-P_{ref}\left(n\right)\right|}{P_{ref}\left(n\right)}\right]\times 100\%$$

where *P_ref_* is the reference pressure applied by the force gauge, *P_sens_* is the pressure measurement taken by the sensor, *n* is the sample number of a specific test run, and *N* is the total number of samples taken.

### 2.4. Repeatability Test

The ability of a sensor to provide the same response with the same amount of applied pressure during repeated measurements is known as its repeatability. Loading was gradually applied onto each sensor by the force gauge and this procedure was repeated five times. The coefficient of variation (CV%) of the sensor reading denotes the repeatability of the measurements.

### 2.5. Time Dependency Test

Drift is the change in the output signal when a constant pressure is applied over a period of time and calculated by comparing the pressure value at time t with the initial steady value (data at 30 s after pressure application is regarded as being in the steady state). The pressure drift (*D_P_*) in percentage is calculated as in Equation (4). The time dependency was tested by applying loads of 30 gram-force (gf) and 80 gf onto the sensor for 5 h (if applicable).

(4) $$D_{P}=\frac{\left(P_{t}-P_{i}\right)}{P_{i}}\times 100\%$$

where *P_t_* is the pressure at time t over which the pressure is applied, and *P_i_* is the pressure at the initial moment after pressure application.

## 3. Results and Discussion

### 3.1. Effect of Sensor Attachment

Adding an attachment to the surface of a sensor can properly support the sensor, enhance the uniformity of the force exerted onto the surface of the sensor, and minimize sensor deformation when mounted on a soft surface. Preliminary studies have found that measurements on a soft surface tends to provide a lower voltage reading than on a rigid surface which can be attributed to the sagging of the surface of the contacting object. This section aims to investigate whether applying an attachment onto the sensor surface would reduce its inaccuracy.

**Figure 6:**
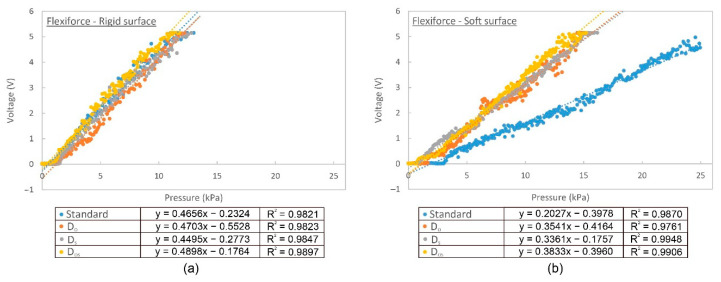

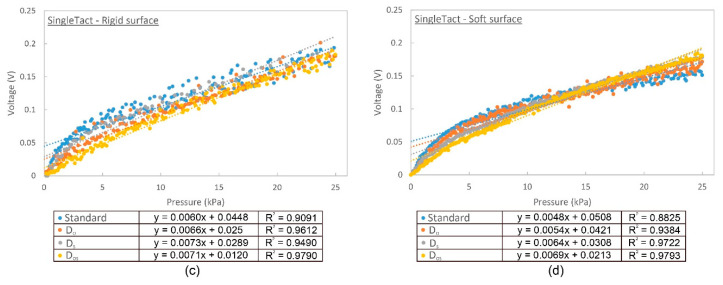
X-axis corresponds to applied pressure converted from force gauge reading. Y-axis corresponds to sensor reading in voltage. (**a**) FlexiForce sensor calibration curve on rigid surface, (**b**) FlexiForce sensor calibration curve on soft surface (silicone of 30 Shore OO hardness), (**c**) SingleTact sensor calibration curve on rigid surface, (**d**) SingleTact sensor calibration curve on soft surface (silicone of 30 Shore OO hardness).

Four sensor conditions were used for the calibration of both the rigid and soft (30 Shore OO) surfaces by establishing a relationship between the applied load and sensor output. The calibration curves for FlexiForce sensor with the measured voltage in the y axis and applied pressure in the x axis are shown in Figure 6a,b. With reference to the distribution of the data points, it can be found that the linearity of the FlexiForce data is excellent at rigid surface (Figure 6a). Its coefficient of determination (R^2^) is higher than 0.97 irrespective of the incorporation of an attachment disc. The linearity of sensor is not evaluated on the soft testing surface since the viscoelasticity property of soft materials can be nonlinear. For the rigid contacting surface, it can be observed in Figure 6a that the calibration curves of the four sensor attachment conditions do not show remarkable differences. Therefore, this suggests that an additional attachment is not necessary when the sensor is used for measurements of a rigid and flat surface. However, if the contacting object is soft, there is a remarkable difference among the four testing conditions as shown in Figure 6b. For the FlexiForce sensor, the slope for the Standard condition is remarkably smaller (i.e., 0.2027) whereas the slope for D_O_, D_S_ and D_OS_ is larger—0.3541, 0.3361, and 0.3833, respectively. This appears that the FlexiForce sensor with the D_OS_ setting results in higher sensitivity. In addition, the smallest detectable pressure for the Standard condition is 3.09 kPa whereas the cases of D_O_, D_S,_ and D_OS_ are just 1.58 kPa, 0.83 kPa, and 0.69 kPa, respectively. The sensors in the Standard and D_O_ conditions were pressed onto a soft contacting object and the soft surface might have deformed. Force was unevenly distributed and concentrated at the edge of the sensor, so a larger force was required to trigger the sensor.

As for the SingleTact sensor, its calibration curves are shown in Figure 6c,d. The linearity of the SingleTact sensor on rigid surface is slightly less than that of the FlexiForce sensor, particularly for the Standard condition in the low pressure range. For the result of rigid surface (Figure 6c), there are considerable differences in the R^2^ among Standard, D_O_, D_S,_ and D_OS_. The R^2^ for D_OS_ is the highest (R^2^ = 0.9790) whilst the R^2^ for Standard is the lowest (R^2^ = 0.9091). When comparing the corresponding calibration curves of the measurements of the rigid and soft surfaces, the discrepancy for Standard is the highest due to the poor rigid support of the sensor and vice versa for D_OS._ This suggests that D_OS_ offers little change in voltage with changes in the surface hardness.

### 3.2. Effect of Surface Temperature of Contacting Object

According to the FlexiForce Sensors User Manual [35], force sensors should be calibrated at the same temperature in which testing occurs. However, they are usually calibrated on an object at room temperature but applied on a surface with a higher temperature, like skin. This section aims to examine whether placing a sensor onto a surface with different temperatures would result in different sensor readings.

**Figure 7:**
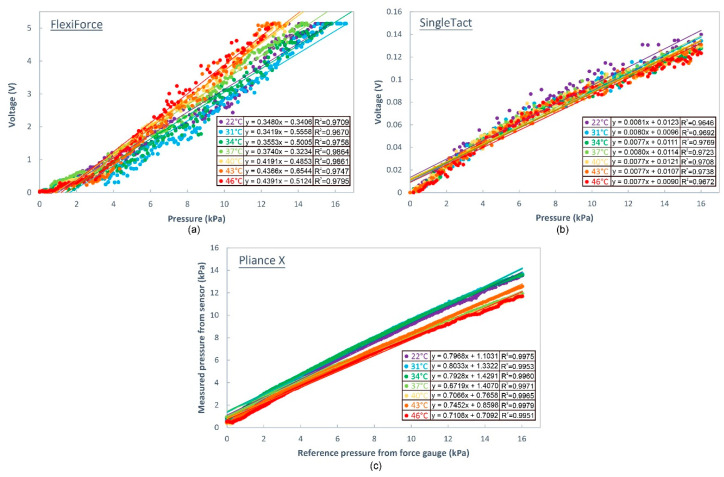
Calibration curves of different sensors measured on soft surface (silicone of 00-30 hardness) maintained at different temperatures: (**a**) FlexiForce sensor, (**b**) SingleTact sensor, and (**c**) Pliance X sensor.

Figure 7 shows the sensor calibration curves of different sensors under various contacting temperatures. The calibration curves of the FlexiForce sensor is shown in Figure 7a, where it can be observed that the slope of the calibration curves increases with the contacting temperature. For every increase of 1 kPa of applied force, the increase in voltage is higher for higher temperature conditions (e.g., 0.348 V at 22 °C but 0.439 V at 46 °C). This suggests that sensitivity of the sensor increases with the contacting temperature.

**Figure 8:**
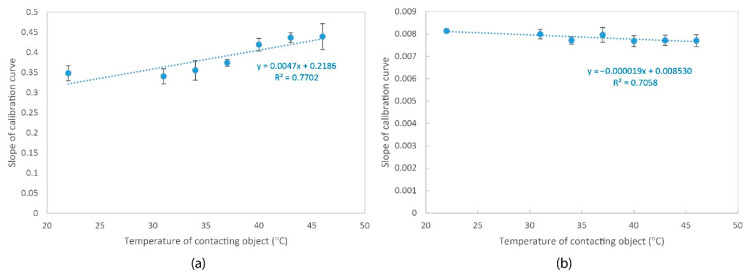

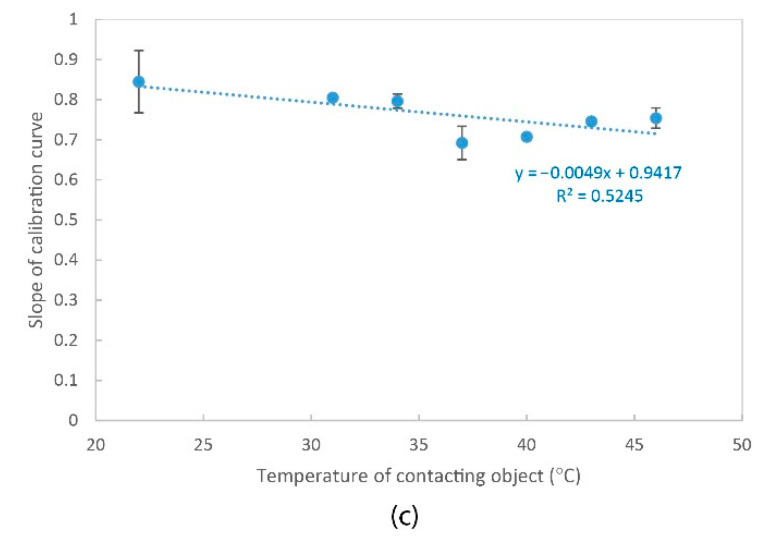
Correlation between slope of calibration curve and temperature of contacting object for (**a**) FlexiForce sensor, (**b**) SingleTact sensor, and (**c**) Pliance X sensor.

A correlation analysis, as shown in Figure 8a, reveals that the slope of the calibration curve is positively and greatly related to the temperature of the contacting object (y = 0.0047x + 0.2186, R^2^ = 0.7702). The independent *t*-test results show that the slope of the calibration curve at a temperature of 22 °C does not show a significant difference with that at a temperature of 31 °C (*p* > 0.05) and 34 °C (*p* > 0.05), but is remarkably lower than that at 37 °C (*p* < 0.05), 40 °C (*p* < 0.05), 43 °C (*p* < 0.05) and 46 °C (*p* < 0.05). This suggests that calibration performed at room temperature can still be used to predict the conditions in which the contacting temperature is as high as 34 °C.

For the SingleTact sensor, the calibration curves, as shown in Figure 7b, are close to each other and overlapping. The correlation analysis, as shown in Figure 8b, shows that the slope of the calibration curve is negatively related to the temperature of the contacting object (R^2^ = 0.7058), thus suggesting that the sensitivity of the sensor decreases with the contacting temperature.

For the Pliance X sensor which is shown in Figure 7c, the calibration curves can be generally divided into two groups based on their slope. The first group consists of the samples with a lower contacting surface temperature (i.e., 22 °C, 31 °C, and 34 °C). Their slope is comparatively larger (ranges from 0.79 to 0.80). The second group includes the samples with a higher contacting surface temperature (i.e., 37 °C, 40 °C, 43 °C, and 46 °C) with a smaller slope (between 0.67 to 0.74). There is a steep change in the slope when the temperature increases from less than 34 °C to over 37 °C. The correlation analysis, as shown in Figure 8c, shows that the slope of the calibration curve has a moderate and negative correlation with the temperature of the contacting object (R^2^ = 0.5245).

Calibration is not carried out for some applications in realistic end-use conditions, and instead, their sensors are calibrated at room temperature only which might cause errors. Next, we quantify the error if the output of the sensor was converted based on a calibration temperature of 22 °C (regardless of the actual used temperature). The results are summarized in Table 3. In general, the percentage error is particularly high when pressure is less than 1 kPa because the measured voltage is close to zero. In addition, there is the trend in which the percentage error increases with the surface temperature of the contacting object. This again suggests that proper calibration based on actual use conditions is important.

Briefly, the repeatability of the sensors is relatively constant with change in the contacting temperature (Table 4). The CV% is extremely high with the FlexiForce sensor when the pressure is less than 1 kPa. However, the CV% is reduced with increases in pressure. The repeatability of the Pliance X sensor is excellent. The average CV% is less than 5% on average. Finally, the repeatability of the SingleTact sensor is more than the FlexiForce but less than Pliance X.

### 3.3. Effect of Hardness of Human Tissue Surrogate

Force and pressure sensors are sometimes used on the interface between skin and apparel to measure the pressure applied by the apparel. As shown in Appendix A, the hardness of skin can vary. This section discusses whether hardness of a contacting object might cause variations in the calibration curve and sensor performance.

**Figure 9:**
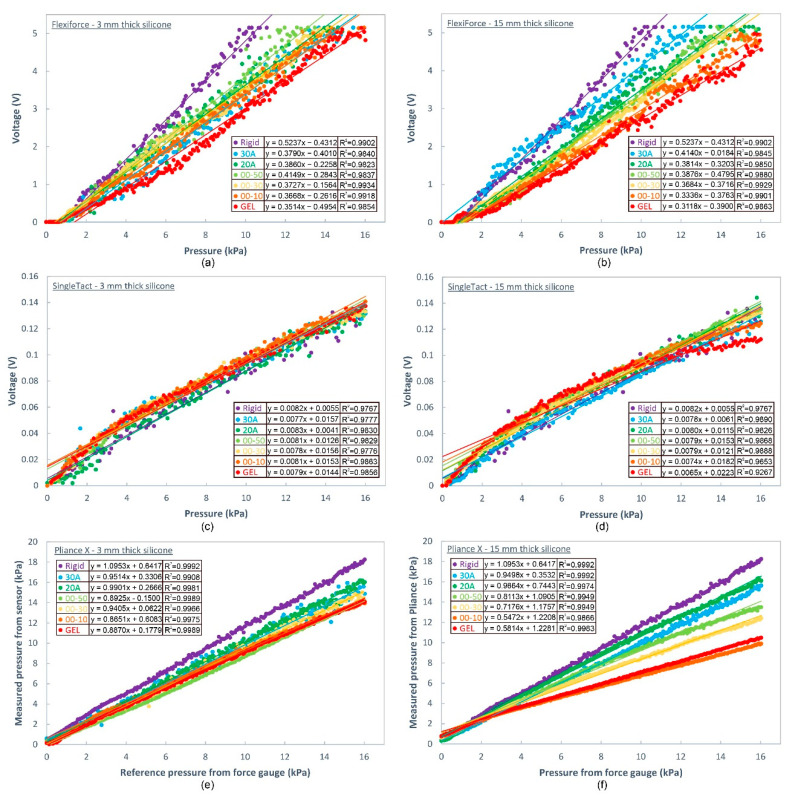
(**a**) FlexiForce sensor calibration curves on rigid and soft (silicone with thickness of 3 mm) surfaces, (**b**) FlexiForce sensor calibration curves on rigid and soft (silicone with thickness of 15 mm) surfaces, (**c**) SingleTact sensor calibration curves on rigid and soft (silicone with thickness of 3 mm) surfaces, (**d**) SingleTact sensor calibration curves on rigid and soft (silicone with thickness of 15 mm) surfaces, (**e**) Pliance X sensor calibration curves on rigid and soft (silicone with thickness of 3 mm) surfaces, and (**f**) Pliance X sensor calibration curves on rigid and soft (silicone with thickness of 15 mm) surfaces.

The FlexiForce and Pliance X sensors have widely spaced calibration curves (see Figure 9), particularly for the silicone samples with a thickness of 15 mm. The steeper curves correspond to a more rigid surface and more gentle curves to a softer surface. For the silicone layer with a thickness of 3 mm, the slope of the calibration curve and hardness of the silicone surface do not have an obvious relationship. Therefore, it can be concluded that the silicone layer with a thickness of 3 mm is not thick enough to show any changes. However, the use of a thicker silicone layer, like the silicone layer with a thickness of 15 mm, shows a progressively larger slope with hardness of the contacting object.

The SingleTact sensor, however, shows that the seven calibration curves are mostly overlapping, except for the curve of the silicone layer labeled “GEL” with a thickness of 15 mm in Figure 9c,d. This implies that the surface hardness has less effect with the use of the SingleTact sensor.

To further investigate the error when the output of the sensor is converted based on the calibration curve of a rigid surface, the percentage error is calculated with reference to Equation (3) and the results are summarized in Table 5. In general, the percentage error is independent of the surface hardness in the samples with a thin layer of silicone (i.e., silicone with a thickness of 3 mm). However, for the samples with a thicker layer of silicone of 15 mm, the percentage error is reduced substantially with increased hardness of the silicone. For the softest material investigated, which is labeled “GEL”, the average percentage error is as high as 39.7% with the FlexiForce sensor, but 18.2% for the SingleTact sensor and 25.8% with the Pliance X sensor. Therefore, this suggests that the sensors should be properly calibrated for use on a soft medium especially the FlexiForce sensor.

In terms of testing repeatability, the CV% of the various testing conditions is summarized in Table 6. The table shows that the repeatability of the sensors does not depend on the surface hardness. In general, the CV% of the Pliance X sensor is low followed by SingleTact whereas FlexiForce provides fewer repeatable results.

### 3.4. Effect of Time on Measurement Accuracy

**Figure 10:**
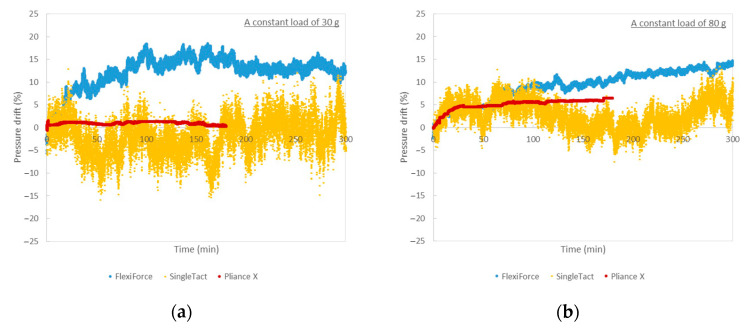
(**a**) Pressure drift of different sensors with time (applied load of 30 gf) and (**b**) Pressure drift of different sensors with time (with applied load of 80 gf).

The sensors were subjected to a constant load for 5 h (the Pliance X sensor was used for only 3 h due to machine configurations) and the changes in pressure with time were measured. Two force levels are presented (30 gf and 80 gf) in random order three times. As shown in Figure 10a,b, the degree of the pressure drift is greater with the FlexiForce sensor and shows an upward drift in pressure with time, particularly for the first hour. Sensor drift may cause problems when high accuracy for a long period of time is required [24]. The pressure drift of the SingleTact sensor fluctuates between the zero points, thus giving positive and negative results intermittently. No obvious trend of the pressure drift can be observed. The pressure drift of the Pliance X sensor is relatively constant throughout the entire testing period.

## 4. Conclusions

A dynamic sensor calibration setup is proposed in this study. The applied pressure is progressively increased and recorded by using both a force gauge and force/pressure sensor. The sensor is calibrated by establishing a relationship between the applied pressure and the corresponding reading. The performance of the sensors including accuracy, repeatability and drift is measured. Testing is conducted with a focus on the properties of the contacting surface. Silicone layer samples with different thickness and hardness are attached to the calibration setup to simulate different skin conditions. The temperature of the skin surrogate is also controlled to simulate the skin temperature under different activity levels. The advantages of this dynamic sensation calibration set-up include:

- Ensuring that force is applied in the same location of the sensor, thus enhancing repeatability of the calibration process;
- Ensuring that force is applied perpendicular to the plane of the sensor;
- Simplicity of the set-up;
- User-friendliness;
- Efficiency in calibration time as compared to dead-weight calibration procedures;
- Measuring the desired pressure range; and
- Simulating the actual wear conditions where the contacting object is subjected to dynamic pressure changes, e.g., breathing and body movement.

Three commercially available sensors, the FlexiForce, SingleTact and Pliance X sensors, are used to determine the influence of the properties of the contacting surface. The FlexiForce and SingleTact sensors are relatively thin, so the effect of sensor attachment is studied. The results show that the plotted slope of the calibration curve is the smallest for the Standard condition where no support is provided to the sensor surface, whereas D_O_**_S_** (i.e., aluminum discs are adhered to both sides of the sensor) can improve the sensitivity of the sensor. Therefore, adhering discs on both sides of the sensor surface can ensure that force is evenly distributed on the sensor and prevent sensor deformation.

The effect of the surface temperature and hardness has also been evaluated. It is found that the change in sensor output depends on the underlying surface and the different sensors are affected to different extents. The calibration curves of the surfaces of different temperatures and hardness are compared. The curves for the FlexiForce and Pliance X sensor spread widely, thus suggesting that they are more sensitive to the changes in temperature and hardness of the contacting surface. The sensitivity of the FlexiForce sensor increases with contacting temperature and surface hardness. The sensitivity of Pliance X sensor, on the other hand, decreases with contacting temperature but increases with surface hardness. As for the SingleTact sensor, most of its calibration curves are close to each other and overlapping. The sensitivity of this sensor decreases with the contacting temperature and surface hardness has less impact on its accuracy.

The percentage error is also calculated in which measurements are converted based on the “standard” calibration condition. When measurements are taken on the surfaces with a higher temperature (refer to calibration curve at temperature of 22 °C), the FlexiForce, SingleTact, and Pliance X sensors all obtain a similar percentage error. When the measurement is performed on a soft surface (refer to calibration curve obtained with rigid surface), the percentage error is particularly high for the FlexiForce sensor. This suggests that individual surface calibrations are necessary when the FlexiForce sensor is used on softer material.

In terms of sensor repeatability, the performance of Pliance X is generally better when tested on surfaces with different temperatures and hardness. In contrast, the repeatability of FlexiForce is comparatively lower.

Finally, these three sensors are subjected to pressure for a longer period of time (5 h for FlexiForce and SingleTact, and 3 h for Pliance X). SingleTact and Pliance X provide a lower pressure drift%, thus implying that they are more suitable for measurements that require a prolonged period of time.

## Figures and Tables

**Table 1:** Sensor specifications.

Company Name	Model Number	Sensor Type	Principle	Operating Range	Active Sensing Area Based on Diameter (mm)	Thickness (mm)
Tekscan (USA)	FlexiForce A201-1	Force sensor	Electrical resistance	0–454 g	9.53	0.203
SingleTact (USA)	S8-10N	Force sensor	Capacitance	0–1000 g	8	0.35
Novel.de (Germany)	Pliance X	Pressure sensor	Capacitance	0.5–60 kPa	10	0.95

**Table 2:** Specifications of silicone.

Product Name	Shore Hardness
Dragon Skin ^TM^ 30	30 Shore A
Dragon Skin ^TM^ 20	20 Shore A
Ecoflex™ 00-50	50 Shore OO
Ecoflex™ 00-30	30 Shore OO
Ecoflex™ 00-10	10 Shore OO
Ecoflex™ GEL	35 Shore OOO

**Table 3:** Percentage error for different sensors when in contact with surface maintained at different temperatures with calibration at room temperature.

Temperature of Contacting Object	FlexiForce ^A^						SingleTact ^A^						Pliance X ^B^					
	0 kPa ≤ P < 1 kPa	1 kPa ≤ P < 5 kPa	5 kPa ≤ P < 9 kPa	9 kPa ≤ P < 13 kPa	13 kPa ≤ P < 17 kPa	Average ^C^	0 kPa ≤ P < 1 kPa	1 kPa ≤ P < 5 kPa	5 kPa ≤ P < 9 kPa	9 kPa ≤ P < 13 kPa	13 kPa ≤ P < 17 kPa	Average ^C^	0 kPa ≤ P < 1 kPa	1 kPa ≤ P < 5 kPa	5 kPa ≤ P < 9 kPa	9 kPa ≤ P < 13 kPa	13 kPa ≤ P < 17 kPa	Average ^C^
31 °C	204.5	27.2	24.5	9.2	4.9	17.4	359.1	29.9	5.8	4.7	10.2	13.9	163.1	30.4	3.8	6.1	13	12.1
34 °C	192.7	22.2	14.2	4.7	3.2	12.3	329	26.5	4.4	5.2	10.4	11.8	198.2	33.8	4.1	6.5	13.3	13.4
37 °C	215.4	20.4	3.5	9.1	10.4	11.5	365.8	26.9	8	3.5	8	12.1	213.3	22.5	10	19.7	24.5	14.4
40 °C	178.1	15.9	11.1	18.5	16.3	15.3	305.9	24.8	6.4	4.8	10.6	12.1	45.6	6	15.4	21.5	25.9	19.2
43 °C	171.2	19.3	7.8	20.3	17.1	16	327.8	25.1	6	6.9	10.8	12.8	81.6	8.9	10.6	17.3	20.8	17.2
46 °C	182.3	13.5	18.6	23.3	-	17.6	394.6	42	4.4	6.4	13	17.4	52.9	5.5	15.3	21.6	26.4	14.4

^A^ The voltage reading measured in each condition is converted into a pressure based on surface calibration curve at temperature of 22 °C as shown in Figure 7. (i.e., FlexiForce: *Voltage = 0.348 Pressure − 0.3406*; SingleTact: *Voltage = 0.0081 Pressure + 0.0123*). The converted pressure is compared with the reference pressure based on Equation (2). ^B^ The measured pressure is directly compared with the reference pressure from the force gauge with Equation (2). ^C^ Average percentage error between 1 and 17 kPa is calculated.

**Table 4:** Repeatability of different sensors measured at different contacting temperatures (Coefficient of variation for repeated measurements of sensor reading).

Temperature of Contacting Object	FlexiForce						SingleTact						Pliance X					
	0 kPa ≤ P < 1 kPa	1 kPa ≤ P < 5 kPa	5 kPa ≤ P < 9 kPa	9 kPa ≤ P < 13 kPa	13 kPa ≤ P < 17 kPa	Average ^A^	0 kPa ≤ P < 1 kPa	1 kPa ≤ P < 5 kPa	5 kPa ≤ P < 9 kPa	9 kPa ≤ P < 13 kPa	13 kPa ≤ P < 17 kPa	Average ^A^	0 kPa ≤ P < 1 kPa	1 kPa ≤ P < 5 kPa	5 kPa ≤ P < 9 kPa	9 kPa ≤ P < 13 kPa	13 kPa ≤ P < 17 kPa	Average ^A^
22 °C	120.5	19.3	9.2	6	3.2	11.2	57	14.4	5	3.1	2.3	7.3	19	5.1	1.6	1.4	1.3	2.3
31 °C	138	26.7	8.4	4	2.6	13.4	120	12.9	4.5	3.7	3.3	7.2	9.7	3	1.4	1.2	0.8	1.5
34 °C	175.6	40	13.1	6.1	4.2	21	107.4	13	3.6	3	2.4	6.3	28.2	8.6	3.9	2.4	1.3	3.7
37 °C	57.7	11.9	4.6	3.3	2.2	6.6	57.3	9.2	4.8	2.7	3.5	5.6	34.8	10.8	4.1	2.6	1.5	4.7
40 °C	59	14.7	6	4.2	4.1	8.9	68.8	9.9	6.4	3.7	2.6	6.3	27	6	3	2.5	1.7	3.1
43 °C	80.5	43	6.2	4.7	-	21	82.2	16.4	7.3	4.4	4.6	9.8	36.9	4.1	2	1.3	0.9	1.9
46 °C	57.2	24.2	6.9	6.5	-	14.5	123.5	26.8	9.1	4.2	3.4	14.7	63.4	14.1	4.9	3.8	2.5	2.3

^A^ Average CV between 1 and 17 kPa is calculated.

**Table 5:** Percentage error of different sensors when in contact with surfaces with different hardness with calibration on rigid surface.

		FlexiForce ^A^						SingleTact ^A^						Pliance X ^B^					
		0 kPa ≤ P < 1 kPa	1 kPa ≤ P < 5 kPa	5 kPa ≤ P < 9 kPa	9 kPa ≤ P < 13 kPa	13 kPa ≤ P < 17 kPa	Average ^C^	0 kPa ≤ P < 1 kPa	1 kPa ≤ P < 5 kPa	5 kPa ≤ P < 9 kPa	9 kPa ≤ P < 13 kPa	13 kPa ≤ P < 17 kPa	Average ^C^	0 kPa ≤ P < 1 kPa	1 kPa ≤ P < 5 kPa	5 kPa ≤ P < 9 kPa	9 kPa ≤ P < 13 kPa	13 kPa ≤ P < 17 kPa	Average ^C^
3 mm	30A	113.5	25.3	29.8	25.7	27	27	88.2	38.2	18.1	6.2	3.2	17	104.8	10.3	4.4	3.6	4	5.7
	20A	112.1	17.1	18.9	22.2	25.1	19.7	151.7	33.8	5.8	3.4	3.8	11.6	49.6	8.9	3.9	2.1	1.8	4.2
	00-50	111.7	12.5	17.4	17.6	21.1	15.8	119.7	28.5	19.3	8.2	2.3	14.6	34.5	14.4	15.2	12.2	11.1	13.3
	00-30	113.4	11.5	20.2	24.9	23.4	19.2	85.7	36.4	21.1	6.9	2	16.7	15.5	4.7	5.7	4.5	6.3	5.3
	00-10	112.7	19.7	25.6	26.2	28.4	24.5	90.4	44	20.6	10.6	3.1	19.6	45.2	7.7	2.5	6.7	11.2	7
	GEL	113.1	30.1	41.4	34.2	31.6	34.5	83	34.9	17.9	6.3	1.6	15.2	35.9	5.6	7.5	9	11.2	8.3
15 mm	30A	119	11	9.5	13.4	18.8	11.2	328.7	44.3	4.5	3.4	7.9	8.8	81.9	8.8	1.1	1.6	2.9	3.6
	20A	113.1	26.1	23.5	22.9	28.1	24.7	170.2	20.6	13.4	4.5	2.6	10.4	59.4	25.4	12	6.8	1.8	11.6
	00-50	111.7	35.9	26.5	26.2	27.9	29.3	721.9	28.4	14.9	7.1	2.2	13.2	149.7	18.8	2.2	7.2	14.1	10.6
	00-30	113	20.7	31.9	28.6	27.1	27.1	140.3	17.4	12.9	3.8	2.1	9.1	121.2	16.2	7.8	16.9	22	15.7
	00-10	115.1	32.2	36.8	34.9	35.5	34.8	129.3	28	18	6.3	7.6	15	158.3	14	25.9	34	37.7	27.9
	GEL	121.9	38.1	41.8	39.2	39.6	39.7	99.8	30.2	18.6	4.8	18.7	18.2	188.3	15.8	22.9	30.6	34	25.8

^A^ The voltage reading measured for each condition is converted to pressure value based on calibration curve of rigid surface. (i.e., FlexiForce: Voltage = 0.5237 Pressure − 0.4312; SingleTact: Voltage = 0.0082 Pressure + 0.0055). The converted pressure value is compared with the reference pressure value by using Equation (2). ^B^ The measured pressure is directly compared with the reference pressure from force gauge with Equation (2). ^C^ Average percentage error between 1 and 17 kPa is calculated.

**Table 6:** Repeatability of different sensors measured on surfaces with different hardness. (Coefficient of variation for repeated measurements of sensor reading).

		FlexiForce						SingleTact						Pliance X					
		0 kPa ≤ P < 1 kPa	1 kPa ≤ P < 5 kPa	5 kPa ≤ P < 9 kPa	9 kPa ≤ P < 13 kPa	13 kPa ≤ P < 17 kPa	Average ^A^	0 kPa ≤ P < 1 kPa	1 kPa ≤ P < 5 kPa	5 kPa ≤ P < 9 kPa	9 kPa ≤ P < 13 kPa	13 kPa ≤ P < 17 kPa	Average ^A^	0 kPa ≤ P < 1 kPa	1 kPa ≤ P < 5 kPa	5 kPa ≤ P < 9 kPa	9 kPa ≤ P < 13 kPa	13 kPa ≤ P < 17 kPa	Average ^A^
Hard		63.3	12.1	6.6	3.2	-	9.3	63	20.1	6.7	5.2	5.2	12.4	8.8	3.5	2.3	1.7	1.3	2.2
3 mm	30A	79	25.9	8.2	6.7	3.6	14.3	27.6	9.5	3.4	3.4	3.4	5.1	5.4	9.6	9.1	5.6	5.2	7.6
	20A	63.9	29.6	10.1	6	3.5	17.3	119.1	32.2	6.8	3.3	2.5	10.9	20.3	5.5	4.9	4.1	2.8	4.3
	00-50	112.8	14	10.5	6	0	10.5	98.4	14.5	4.6	3.1	2.5	6.5	21.4	3.1	1.3	1.1	0.9	1.5
	00-30	102.7	14.2	7	5	2.2	8.5	60.9	12.5	3.9	3.3	2.1	5.5	46.2	13.1	7.9	5.4	4.2	7.3
	00-10	91.1	19.6	5.8	6.7	4.8	10.1	37.7	7.1	3.8	2.9	2.1	4	25.7	5.6	3.4	2.6	2.1	3.3
	GEL	44.7	18.9	9.4	5.6	3.5	9.9	72.8	12.9	6.1	3.7	2.8	6.4	138.4	17.2	4.8	2.8	1.7	6.1
15 mm	30A	59.5	14.5	7.4	4	-	9.3	117.9	10.3	3.2	2.9	2.3	4.9	15.2	6.6	3.5	2.6	2	2.2
	20A	88.2	18.3	7.2	6.7	3.1	11.1	41.5	11.6	3.4	3.2	2.6	5.6	10.1	3.2	1.1	1.4	1.1	3.5
	00-50	56.7	26.2	6.5	5.6	6.2	12.4	18.7	8.2	4.1	2.5	2	4.2	14.2	4.4	2.2	2	1.4	1.7
	00-30	104.2	16.6	9	5.3	2.6	9.4	42.9	9.3	4.3	3.4	2.3	4.8	15.8	5.9	2.7	2.6	2.3	2.3
	00-10	59.6	19.1	10.6	7.3	5.8	11.2	55.1	10.7	5.7	4.3	3	5.9	17.2	5.8	3.4	2.4	1.8	3.2
	GEL	45.6	39.6	10.7	6.5	4.9	15.6	59.6	10.7	3.6	4	3.4	5.6	19.5	4.8	2	1.5	1.2	3.2

^A^ Average CV between 1 to 17 kPa is calculated.

## Appendix A

**Table A1:** Hardness of Human Body or Human Body Surrogate.

Author	Hardness	Body Area/Simulation Material
Muthu [36]	20 Shore A	Skin
Wettels et al. [37]	20–50 Shore A	Skin
Derler et al. [38]	42.5 Shore A	Lorica^®^ Soft fabric
Romanelli and Falanga [39]	16 Shore A	Leg
	50 Shore A	Lipodermatosclerosis zone of venous ulcers
Wettenschwiler et al. [40]	30 Shore A	Entire body prostheses
Cruz et al. [41]	10–90 Shore A	Breast prostheses
Chanda [42]	30 Shore A/ 10 Shore OO	Skin tissue surrogate
Sim et al. [43]	27 to 84 Shore OO	Skin simulant
Pittar et al. [44]	13.3–28 Shore A	Human scalp
	19 Shore A	Skin simulant
Cabibihan et al. [45]	30–86 Shore OO	Soft artificial hand
Falland-Cheung et al. [46]	0–65 Shore A	Human skin
